# Comparative performance of disability measures

**DOI:** 10.1371/journal.pone.0318745

**Published:** 2025-01-31

**Authors:** Scott D. Landes, Jean P. Hall, Bonnielin K. Swenor, Nastassia Vaitsiakhovich

**Affiliations:** 1 Department of Sociology and Aging Studies Institute, Maxwell School of Citizenship and Public Affairs, Syracuse University, Syracuse, New York, United States of America; 2 Institute for Health & Disability Policy Studies and Research & Training Center on Independent Living, University of Kansas, Lawrence, Kansas, United States of America; 3 The Johns Hopkins Disability Health Research Center, Johns Hopkins School of Nursing, Wilmer Eye Institute, Johns Hopkins School of Medicine, Department of Epidemiology, Johns Hopkins Bloomberg School of Public Health, Baltimore, Maryland, United States of America; 4 Lerner Center for Public Health Promotion, Center for Policy Research, and Department of Sociology, Maxwell School of Citizenship and Public Affairs, Syracuse University, Syracuse, New York, United States of America; National center for chronic and non-communicable diesease prevention and control, CHINA

## Abstract

Researchers and federal agencies are currently discussing the best way to measure disability in US federal surveys. One idea being discussed is expanding/supplementing the question sets commonly used to capture disability status in order to better capture a broader segment of the disabled population. We used data from the 2010–2018 National Health Interview Survey to examine the performance of questions commonly used to measure disability in the US–the ACS-6 and Washington Group Short Set questions–in capturing intellectual and developmental disabilities, mental health disabilities, and physical health disabilities. We found that neither set of disability questions was sufficient to fully capture these disability statuses. We contend that current disability questions used in US population surveys must be expanded/supplemented in order to increase the inclusiveness of disability measurement, and thereby, improve efforts to reduce disparities experienced by the disabled population.

## Introduction

In the United States, 26.8% of adults are disabled [1]. Individuals with disabilities are a highly marginalized population, experiencing socioeconomic disparities such as lower levels of education, higher unemployment rates, and inadequate environmental accessibility [2]. These disadvantages contribute to worse health and mortality outcomes [3, 4]. To address these inequities effectively, it is essential to have accurate and timely disability data. Such data enable the evaluation, planning, and implementation of programs and policies aimed at ensuring equity for all individuals with disabilities.

In October 2023, the US Census Bureau–a principal agency of the U.S. Federal Statistical System that collects data about the nation’s people and economy–announced that it was considering changing the questions used to measure disability status in the American Community Survey [5]. Since 2008, the American Community Survey included a set of questions commonly known as the ACS-6 to measure disability [6]. These questions are aligned with the International Classification of Functioning, Disability, and Health (ICF)–the conceptual framework developed by the World Health Organization, which defines disability as the product of the interaction between impairment, functional limitations, and environmental factors that may act as barriers to participation in social life [7, 8]. In 2011, the Secretary of the Department of Health and Human Services designated the ACS-6 questions as the minimum standard for measuring disability in US federal surveys [9]. They do so by identifying individuals as disabled if they experience a functional limitation in their vision, hearing, mobility, cognition, self-care, or instrumental activities of daily living (IADL) [6].

The Census Bureau’s plan was to change to using the Washington Group Short Set questions (WGSS). The WGSS are a six-question sequence based on the ICF framework, which identifies individuals as disabled if they experience a functional limitation regarding their vision, hearing, mobility, cognition, self-care, or communication. Beyond the difference in the functional limitation categories (IADL prompt only in ACS-6; communication prompt only in WGSS), the WGSS uses a graded response scale (no difficulty, some difficulty, a lot of difficulty, cannot do at all) compared to a dichotomous response format for the ACS-6 (yes, no). Although the WGSS were adopted as a disability measure by the United Nations in 2006 [10], these questions have not been recognized as a standard for measuring disability in the US [11].

The Census Bureau received overwhelming feedback from US disability researchers and advocates [12, 13] regarding the fact that the WGSS is known to severely underestimate the disabled population [14–16]. In response, on February 6, 2024 the Census Bureau halted its plans to change the disability questions [17] and is currently pursuing a whole-of-government approach to deciding next steps [18]. In addition, the White House Office of Science and Technology Policy (OSTP) National Science and Technology Council Subcommittee on Equitable Data (SED) recently established a new federal interagency taskforce, the Disability Data Interagency Working Group (DDIWG) [19]. Co-chaired by representatives from OSTP, the National Institute on Disability, Independent Living, and Rehabilitation Research (NIDILRR), and the Office of Management and Budget (OMB), and inclusive of representatives from the Census Bureau, the DDIWG seeks to “improve the Federal government’s ability to make data-informed policy decisions that advance equity for the disability community” [19].

In the midst of the ongoing discussion regarding disability measurement in the US, a group of disabled and allied researchers proposed a Research Roadmap to move disability measurement forward [20]. As part of this roadmap, they suggested continued use of the ACS-6 questions as opposed to the WGSS questions due to the poor performance of the WGSS. They also recommended expanding/supplementing the ACS-6 questions to better capture people with intellectual and developmental disability (IDD), physical health disability, or mental health disability. This suggestion is based on the concern that neither the ACS-6 nor WGSS questions include prompts for these disability statuses [15, 21, 22]. Using data from the National Survey on Health and Disability (NSHD), an internet based survey of disabled adults aged 18–64 in the US, Hall et al. [15] reported that overall the ACS-6 performed better than the WGSS in capturing disability status, but both question sets underperformed in accurately capturing people with disabilities not mentioned in the question prompts, such as those with disabilities related to chronic illness, mental health or physical health. No research to date empirically examines the percentage of people with IDD, physical health disability, or mental health disability who are captured by the ACS-6 and WGSS in a nationally representative sample of adults.

It may be that the ACS-6 and WGSS fail to estimate people with disabilities in nationally representative data not specifically named in their question prompts. Or, as was reported by Hall et al. in the NSHD data [15], it could be that some people with these disabilities are being captured despite their specific disability status not being included in the prompts. This evidence is crucial in understanding whether the ACS-6 and WGSS questions are sufficiently identifying these disability statuses in nationally representative data, or are in need of being supplemented to better capture these subpopulations of disabled people.

The purpose of this study is to examine the percentage of people with IDD, physical health disabilities, or mental health disabilities who were identified as disabled by the ACS-6 and WGSS questions in nationally representative data.

## Data and methods

### Data and sample

Data are from the 2010–2018 National Health Interview Survey (NHIS) acquired from IPUMS [23]. The NHIS fielded the ACS-6 to a subpopulation of the survey from 2010–2017 (N = 293,442); the WGSS to a subpopulation from 2011–2018 (N = 134,427); and both question sets to a subpopulation between 2011–2012 (N = 24,727).

From 2010–2018 the NHIS also fielded a separate set of activity limitation questions to all adult respondents. These questions ascertained whether the respondent had an activity limitation regarding activities of daily living, IADLs, work, mobility, cognition, or physical, mental, or emotional problems. Respondents who indicated having an activity limitation were then asked to indicate the cause of the activity limitation from a list of 35 conditions. We used the causal condition questions to identify eight disability statuses meeting the definition of disability per the Americans with Disabilities Act (ADA), the US federal civil rights law passed in 1990 that prohibits discrimination against disabled people.

The ACS-6 analysis used data for respondents with an activity limitation and one of the eight ADA defined disability statuses who were fielded the ACS-6 questions in the 2010–2017 data: vision (N = 3,587); hearing (N = 2,375); birth defect (N = 555); intellectual disability (N = 1,110); other developmental disability (N = 622); mental health disability (N = 6,326); cancer (N = 2,105); diabetes (N = 5,470).

The WGSS analysis used data for respondents with an activity limitation and one of the eight ADA defined disability statuses who were fielded the WGSS questions in the 2011–2018 data: vision (N = 2,261); hearing (N = 1,426); birth defect (N = 297); intellectual disability (N = 438); other developmental disability (N = 283); mental health disability (N = 3,788); cancer (N = 1,187); diabetes (N = 3,051).

### Measures

#### ADA defined disability status

The ADA defines disability as a physical or mental impairment that limits one of life’s major activities. Employing a conservative strategy in our analysis, the analytic sample only included adults aged 18 or over with conditions causing activity limitations reported in the NHIS that are clearly identified as disabilities on the ADA website [24]. Although the ADA covers other disabilities not listed as an example on the website, and the Americans with Disabilities Act Amendments Act (ADAAA) of 2008 broadened the definition of disability to include the past history of an impairment or being regarded as having an impairment, this strategy ensured that all individuals included in the analytic sample were disabled per ADA standards. Per this strategy, eight ADA defined disability statuses were included in the analytic sample: hearing disability, vision disability, birth defect, intellectual disability, other developmental disability (NHIS language: “other developmental problem (for example, cerebral palsy)”), mental health disability (NHIS language: depression/anxiety/emotional problem), cancer, and diabetes.

#### ACS-6 disability status

ACS-6 disability status indicated the respondent reported having difficulty in at least one of the following categories of functional limitations: vision, hearing, mobility, cognition, self-care, or instrumental activities of daily living.

#### WGSS disability status

Per the Washington Group guidance on their questions, “disability is defined as those who have a lot of difficulty with or cannot do at all on at least one of the basic functional domains included in the question set” [25]. The National Center on Health Statistics (NCHS) reinforces the Washington Group’s suggested cut point by providing a composite measure (DISAB3_A) of disability status in NHIS data based on this cut point in addition to providing the six individual WGSS measures with the full scale response categories [26, 27]. Thus, WGSS disability status indicated the respondent reported having a lot of difficulty or could not do at all in any of the following categories of functional limitations: vision, hearing, mobility, cognition, self-care, or communication.

## Analytic plan

We report the percentage of adults with a disability as defined by the ADA who were identified as disabled in the ACS-6 and the WGSS questions based on crosstabulations.

To examine whether the results were consistent when limiting analysis to respondents who were fielded both question sets, we conducted sensitivity analysis using only the 2011–2012 cases of adults who reported an activity limitation and one of the eight ADA defined disability statuses, and were asked both the ACS-6 and WGSS questions: vision (N = 1,404); hearing (N = 848); birth defect (N = 203); intellectual disability (N = 362); other developmental disability (N = 215); mental health disability (N = 2,321); cancer (N = 707); diabetes (N = 2,042). Full results from these crosstabulations are provided in S1 Appendix.

As our aim was to understand the performance of the ACS-6 and WGSS in capturing ADA defined disability statuses, and the ACS-6 and WGSS questions were not asked of all adults in the years of the study, all analyses were unweighted. Analysis was conducted using STATA 18.0 (College Station, TX).

## Results

**Fig 1:**
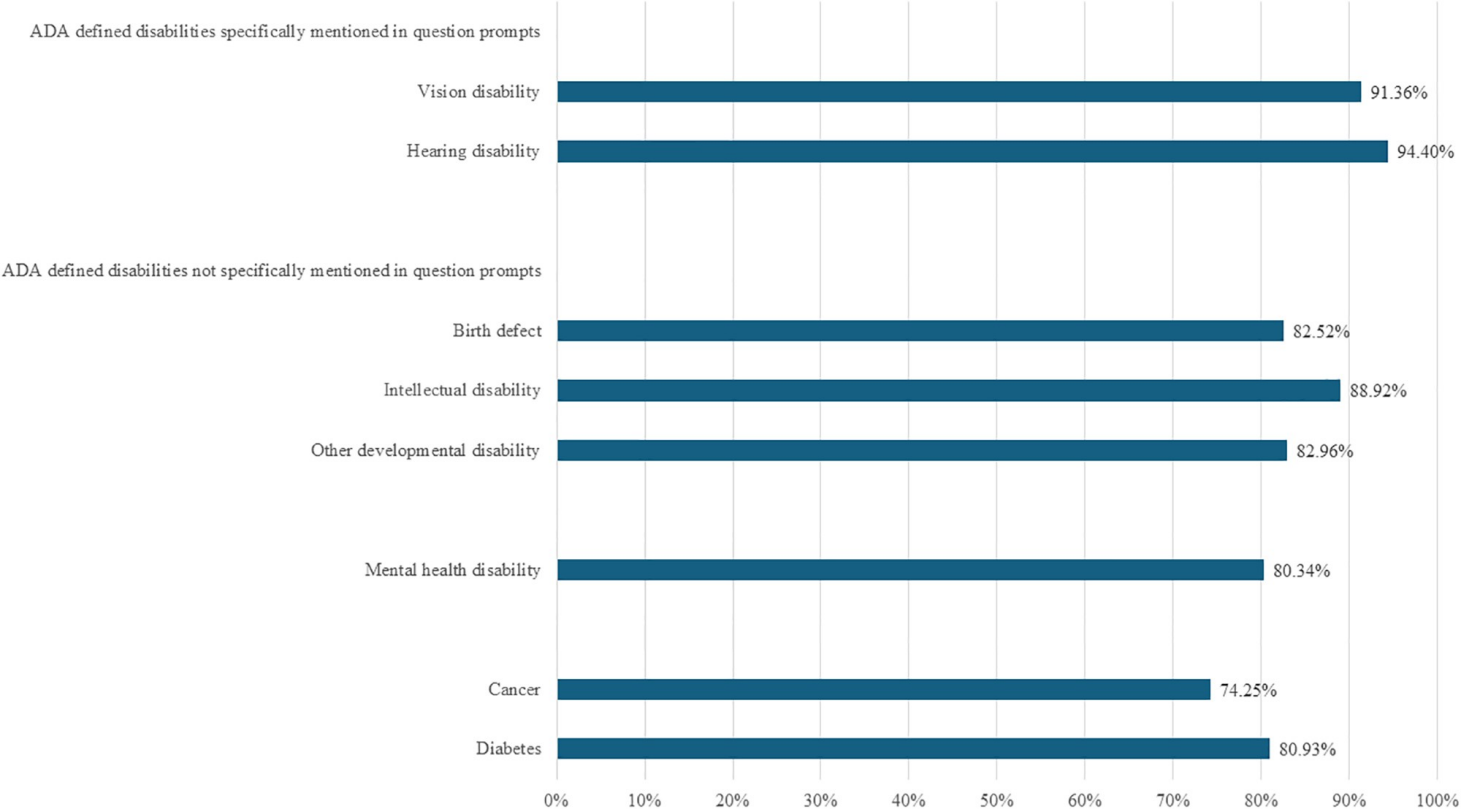
Percentage of ADA defined disability statuses captured by ACS-6, 2010–2017 National Health Interview Survey.

**Fig 2:**
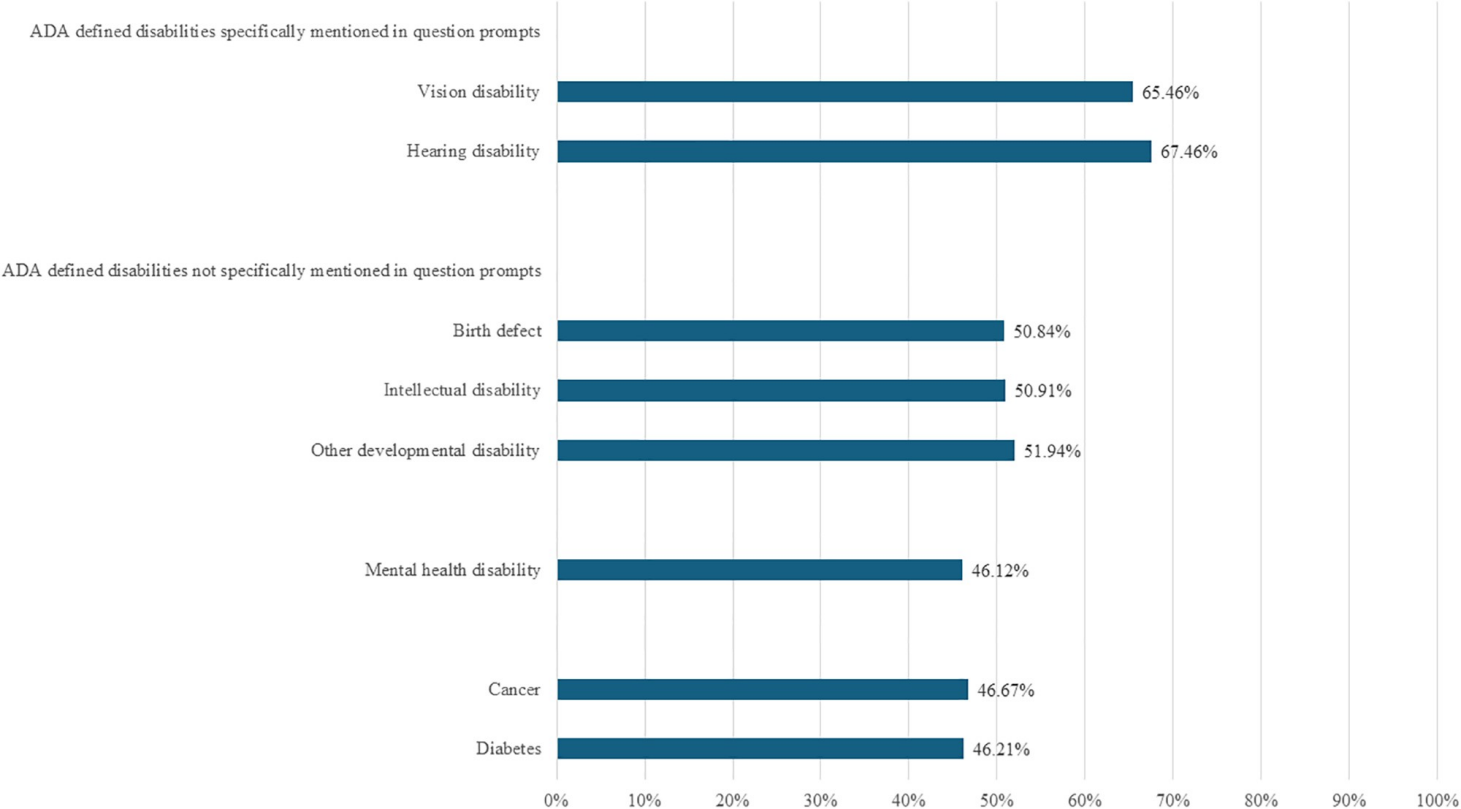
Percentage of ADA defined disability statuses captured by WGSS, 2011–2018 National Health Interview Survey.

Results for the 2010–2017 ACS-6 questions are reported in Fig 1. Results for the 2011–2018 WGSS questions are reported in Fig 2. The percentage of adults with an ADA defined disability who were identified as disabled was highest among adults with a disability status specifically mentioned in the ACS-6 and WGSS question prompts: 91.4% in the ACS-6 and 65.5% in the WGSS for vision disability; 94.4% in the ACS-6 and 67.5% in the WGSS for hearing disability. The percentage of adults with an ADA defined disability who were counted as disabled was comparatively lower among adults with a disability status not specifically named in the ACS-6 and WGSS question prompts: 82.5% in the ACS-6 and 50.8% in the WGSS for birth defect; 88.9% in the ACS-6 and 50.9% in the WGSS for intellectual disability; 83.0% in the ACS-6 and 51.9% in the WGSS for other developmental disability; 80.3% in the ACS-6 and 46.1% in the WGSS for depression/anxiety/emotional disability; 74.3% in the ACS-6 and 46.7% in the WGSS for cancer; and 80.9% in the ACS-6 and 46.2% in the WGSS for diabetes.

Sensitivity analysis of the 2011–2012 data revealed similar percentages to those reported in Figs 1 and 2. Full results are reported in S1 Appendix.

## Discussion

Overall, ADA defined disability categories that are specifically indicated in the ACS-6 and WGSS question prompts were more likely to be captured by these question sets. ADA disability statuses not specifically indicated in the ACS-6 and WGSS question prompts were less likely to be captured. Between the two question sets, the ACS-6 outperformed the WGSS questions across all eight ADA defined disability statuses included in the study.

These results support two suggestions recently made by disabled researchers and allies regarding the path forward to more inclusive and equitable disability measures [20]. First, adding to the stable of research documenting the ACS-6 outperforms the WGSS in identifying disability status [15, 28, 29], results from this study provide further evidence that the ACS-6 performs better than the WGSS in capturing disabilities that are specifically named in these questions. Second, results provide empirical evidence that both the ACS-6 and the WGSS questions fail to capture a large percentage of disabled people with IDD, mental health disability, and physical health disability.

As the agenda proposed in the Research Roadmap [20] outlines, the long-term goal in equitable disability measurement should be the creation of new and more inclusive disability measures. This goal can only be accomplished via robust collaboration between federal partners and the US disability community. But, until this long-term goal is met, results from this study support the shorter and mid-range goals identified in the Research Roadmap of using the ACS-6 as opposed to WGSS in US federal surveys, but expanding/supplementing the ACS-6 questions to include specific prompts for IDD, physical health, and mental health disability. Failure to do so will result in a continued inability to accurately identify specific subpopulations of disabled people in federal population health surveys.

Our analysis only examined the percentages of respondents with eight disabilities who were captured in the ACS-6 and WGSS in the NHIS. It may be that other disability statuses not examined in this study that are and are not specifically named in the prompts for ACS-6 and WGSS questions are associated with different percentages. In addition, these question sets may perform differently in other surveys. It is important to realize that as the NHIS questions regarding vision disability, hearing disability, birth defect, intellectual disability, other developmental disability, mental health disability, cancer, and diabetes were self-reported. Thus, they may not have captured all respondents with these disability statuses. Finally, and possibly most importantly, the disability measures fielded in the NHIS as well as many other US federal surveys only capture disabled people who have an activity or functional limitation. As not all disabled people have these limitations [30], results from this study are only descriptive of disabled people with activity or functional limitations. Together, these limitations underscore the need for more inclusive disability measures in US federal surveys [20]. As the focus of this study was on disability measurement in the NHIS, results cannot be generalized beyond this survey or the US.

## Conclusion

The National Institute on Minority Health and Health Disparities (NIMHD) designated disabled people as a population with health disparities on September 26, 2023 [31]. This designation will likely result in increased emphasis on research aimed at better understanding and reducing the health disparities experienced by disabled people. In the midst of this important milestone, it is imperative to remember that efforts to reduce disparities among disabled people, as well as any other health disparities population, rely on inclusive and comprehensive measures [32]. Based on evidence from our study, any hope for understanding and reducing health disparities among disabled people necessitates supplementing/expanding current disability questions to better capture this population.

## Supporting information

S1 Appendix Percentage of ADA defined disability statuses captured by ACS-6 and WGSS questions, 2011–2012 National Health Interview Survey. (TIF)
