# Supplementary figures and images for: Comparative performance of disability measures
Source: PLoS One. 2025 Jan 31;20(1):e0318745. doi: 10.1371/journal.pone.0318745 (PMC11785326; doi:10.1371/journal.pone.0318745)

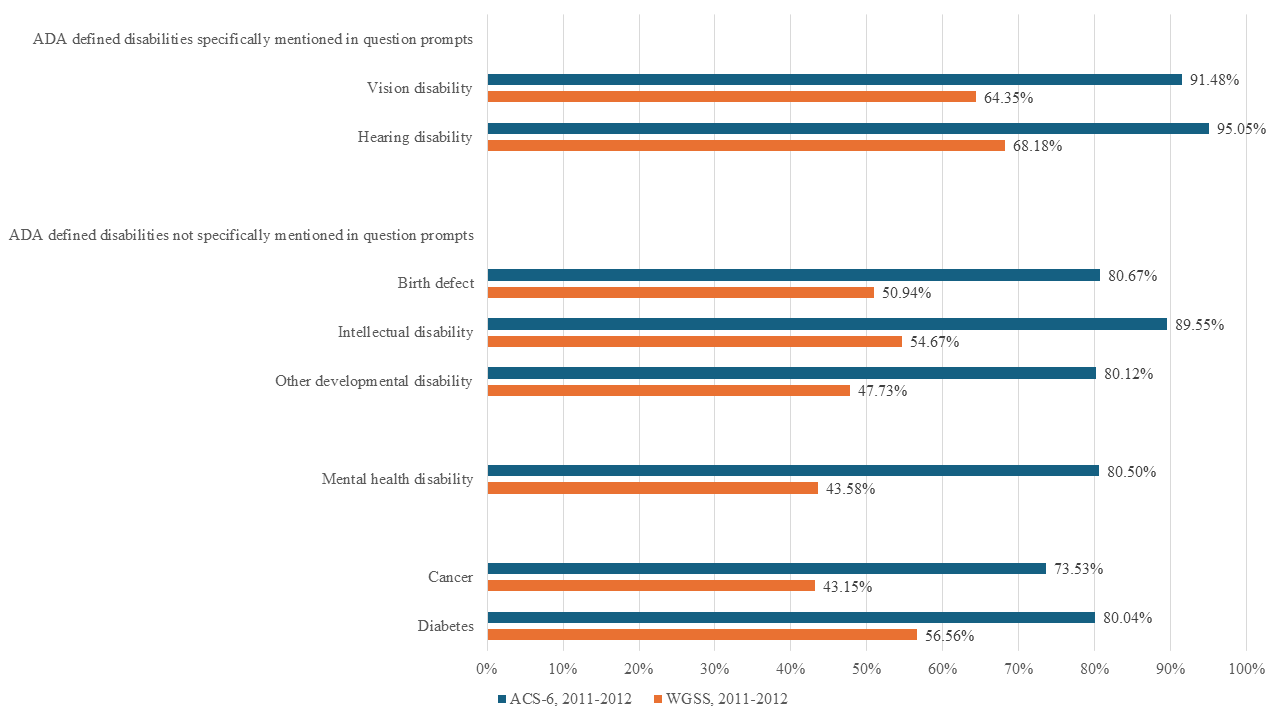

Supplement: S1 Appendix — (TIF) [file pone.0318745.s001.tif]
